# Genomic and integrative based progression biomarker discovery in adult sepsis: toward clinical stratification and precision medicine

**DOI:** 10.1016/j.aicoj.2026.100133

**Published:** 2026-08-17

**Authors:** Silvia Gonzalez-Barbuzano, Eva Suarez-Pajes, Miguel Bardají-Carrillo, Eduardo Tamayo, Tamara Hernandez-Beeftink, Carlos Flores

**Affiliations:** a Research Unit, Hospital Universitario Nuestra Señora de Candelaria, Instituto de Investigación Sanitaria de Canarias (IISC), Santa Cruz de Tenerife, Spain; b Department of Anaesthesiology and Critical Care, Hospital Clínico Universitario de Valladolid, Valladolid, Spain; c Centro de Investigación Biomédica en Red de Enfermedades Infecciosas (CIBERINFEC), Instituto de Salud Carlos III, Madrid, Spain; d Biomedicine Group in Critical Care (BioCritic), Valladolid, Spain; e Departamento de Cirugía, Facultad de Medicina, Universidad de Valladolid, Valladolid, Spain; f Instituto de Investigación Sanitaria de Valladolid (IBioVALL), Valladolid, Spain; g CIBER de Enfermedades Respiratorias (CIBERES), Instituto de Salud Carlos III, Madrid, Spain; h Genomics Division, Instituto Tecnológico y de Energías Renovables (ITER), Santa Cruz de Tenerife, Spain; i Facultad de Ciencias de la Salud, Universidad Fernando Pessoa Canarias, Las Palmas de Gran Canaria, Spain

**Keywords:** Biomarker, Sepsis, Prognostics, Multi-omics

## Abstract

Sepsis is a life-threatening syndrome characterized by a heterogeneous host response to infection that remains a major cause of mortality worldwide. Current clinical scoring systems capture organ dysfunction but fail to reflect the underlying biological diversity, limiting their utility for patient stratification and targeted therapy. This review provides a comprehensive overview of molecular biomarker approaches used to predict sepsis course and prognosis in adult patients, covering genetic, transcriptomic, proteomic, and integrative strategies up to May 2026. Here, we summarize findings from genetic association studies, along with analyses based on polygenic risk scores to aggregate genetic effects, Mendelian randomization, and rare-variant sequencing approaches. We also review transcriptomic and proteomic strategies for endotyping, and diagnostic and prognostic discrimination. Lastly, we discuss how multi-omics integration is emerging as a promising framework to assist in distinguishing causal therapeutic targets from non-causal biomarkers. We also address the challenges that still constrain clinical translation towards precision medicine.

## Background

Sepsis is a life-threatening organ dysfunction caused by a dysregulated host response to infection and constitutes a major global health problem [1]. The estimated hospital incidence is 189 adult patients with sepsis per 100,000 person-years, with a mortality rate of 26.7%, nearly one in four patients does not survive [2]. Clinically, sepsis can progress from early organ dysfunction to severe forms such as septic shock (characterized by profound circulatory, cellular, and metabolic alterations), and may culminate in multiple organ dysfunction syndrome, with a mortality rate exceeding 40% [1,3].

Clinical scoring systems, such as sequential organ failure assessment (SOFA) [4], quick SOFA (qSOFA) [5], the Simplified Acute Physiology Score (SAPS) [6] or Acute Physiology and Chronic Health Evaluation (APACHE) [7], provide practical instruments for risk stratification, and bedside evaluation. Although routinely used in clinical practice, they offer limited insight into the biological heterogeneity and molecular mechanisms driving disease progression [1,8]. Patients with identical SOFA scores can exhibit markedly divergent immune profiles and outcomes, indicating that current scores measure organ dysfunction but fail to capture the underlying biological diversity [9].

To fill this gap, there is growing interest in identifying genetic and other molecular biomarkers that reflect the temporal and biological complexity of sepsis, capturing the dynamic and heterogeneous host response [10]. The host immune response evolves from an initial hyperinflammatory phase, driven by a cytokine storm and endothelial activation, towards a state of immunosuppression or immunoparalysis characterized by T-cell exhaustion, reduced monocyte HLA-DR expression, and increased susceptibility to secondary infections [11]. Although individual inflammatory diagnostic biomarkers, such as C-reactive protein (CRP), procalcitonin (PCT), presepsin (sCD14-ST), and interleukin-6 (IL-6) have improved early detection of infection and sepsis, their ability to predict disease progression and outcomes remain limited [12,13]. Recent evidence suggests that the addition of progression biomarkers, including mid-region proadrenomedullin (MR-proADM), angiopoietin-2, soluble urokinase plasminogen activator receptor (suPAR) and specific microRNAs (miRNAs), to clinical assessment significantly enhances prognostic accuracy beyond baseline diagnostic biomarkers alone [[14], [15], [16], [17]].

The early molecular characterization is beginning to inform treatment selection, with biomarker-guided precision immunotherapy stratification demonstrating improved multiorgan dysfunction of patients [18,19]. This review provides a comprehensive and up-to-date overview of the most relevant genetic, transcriptomic, proteomic, and integrative approaches used up to May 2026 to identify predictors of the course and prognosis of sepsis.

## Host genetics approximations to identify molecular biomarkers

Genetic biomarkers represent units of genomic variation and are measurable characteristics of DNA or RNA with known genomic coordinates. These include single nucleotide polymorphisms (SNPs), mutations, insertions or deletions, or structural variations. They could be associated with susceptibility, progression, or therapeutic response [20]. Epidemiological studies indicate that susceptibility to and progression of infectious diseases have a significant hereditary component. For example, twin-study heritability for SARS-CoV-2 susceptibility and severity were estimated at 30–65 % and 41%, respectively [21,22].

The genetic architecture of sepsis has been primarily explored through candidate gene studies focusing on individual variants in genes encoding pattern recognition receptors (PRRs), cytokines, coagulation regulators, and innate humoral immunity proteins [23]. Genetic variation in Toll-like receptors (TLR) and their co-receptors modulate pathogen recognition. *TLR4*, *TLR2, CD14*, and *NOD2* have been linked to sepsis risk in several studies, although showing inconsistent association across them [[24], [25], [26]]. Candidate SNPs in pro- and anti-inflammatory cytokine genes, such as *TNF*, *IL6*, *IL10* and *IL1RN* have been studied extensively, but show small and context-dependent effects. Genetic variants in regulators of coagulation primarily carry prognostic value. Variants in *SERPINE1*, encoding PAI-1, have been associated with sepsis susceptibility and mortality [[27], [28], [29]]. Regarding innate humoral immunity, an *MBL2* variant linked to reduced mannose-binding lectin have been associated with mortality in pneumococcal sepsis [30,31] but a larger cohort study found no association [32].

These candidate-gene studies examples followed a classical hypothesis-driven approach interrogating a limited set of gene variants preselected based on known or presumed biological function. Candidate gene association studies in sepsis have yielded largely inconsistent and non-replicable genetic predictors [33] challenging the interpretation of findings. This inconsistency is prominently a consequence of the winner’s curse effect, where each new genetic association was systematically overestimated in the discovery study [34]. At the time, standardization of methodological and statistical procedures was needed. For that, embracing genome-wide screening approaches has been key to evidence robust genetic predictors of risk and to better understand the polygenic architecture of complex diseases [35].

### Genome-wide association studies

A genome-wide association study (GWAS) is a standard approach that involves thousands of cases (disease affected) and unrelated controls (unaffected) simultaneously interrogating hundreds of thousands of common genetic variants (minor allele frequencies >1% in general population) across the genome. Based on statistical genetic approaches, GWAS identifies genetic coordinates with statistically significant differences in allele frequencies between cases and controls without prior biological assumptions [36,37]. Unlike candidate-gene studies, GWAS enable the identification of new loci, the elucidation of previously unknown pathogenic mechanisms, and reported novel potential therapeutic targets [35].

Despite its potential, few GWAS have focused on sepsis with a small number of studies focusing on critically ill patients to date. There are three sepsis susceptibility GWAS. D'Urso et al. [38] conducted a GWAS in septic shock patients but found no robust genome-wide significant variant. Similarly, Engoren et al. [39] performed a GWAS in sepsis patients, identifying no genome-wide significant loci. More recently, Vaquerizo-Villar et al. [40] applied an explainable artificial intelligence approach to GWAS data in post-surgical sepsis patients, prioritizing susceptibility variants but without external validation.

Four GWAS of sepsis mortality have been conducted to date. Rautanen et al. [41] reported common variants in the *FER* tyrosine kinase gene strongly associated with 28-day survival in adult ICU patients with pneumonia-induced sepsis. However, replication has been controversial, as an independent attempt was unsuccessful [42], whereas Hinz et al. [43] reported a significant association between *FER* and 90-day survival in ICU patients, although this study was limited by its small sample size [33]. Scherag et al. [44] identified a missense variant in *VPS13A* associated with 28-day mortality in adult sepsis patients, along with converging evidence implicating *CRISPLD2*, previously associated with procalcitonin levels, and a chromosome 13 locus. In a study of septic shock patients, Rosier et al. [45] identified variants within the *CISH* gene, involved in the negative regulation of cytokine signaling, strongly associated with mortality (days 7–28). Functional CRISPR-Cas9 evidence and luciferase assays supported its mechanistic role. The fourth GWAS of 28-day sepsis mortality that has been conducted by Hernandez-Beeftink et al. [46], which identified a missense variant in *SAMD9*, encoding a potential mediator of the inflammatory response to tissue injury. More recently, the largest meta-analysis to date was performed by Tan et al. [47]. Contrary to previous GWAS in sepsis, which used clinically characterized cases, the authors leveraged electronic health records to compare 60,314 sepsis cases with 1,464,733 population controls across large biobanks. The study reported four genetic loci, including a missense variant in *SERPINA1*, which encodes alpha-1 antitrypsin and was also associated with 30-day sepsis mortality in the UK Biobank.

A total of three studies has examined sepsis-associated respiratory complications such as the acute respiratory distress syndrome (ARDS). Guillen-Guio et al. [48] identified a variant in *FLT1* associated with sepsis-associated ARDS susceptibility, with functional assays confirming the allele-specific effects on *FLT1* promoter activity. More recently, the largest ARDS GWAS to date identified a variant near *HMGCR*, linking cholesterol metabolism to ARDS risk, although was not nominally significant in two independent validation cohorts despite consistent direction of effects [49]. A GWAS of sepsis-associated acute kidney injury (AKI) prioritized *CHRNA7*, *NR5A*2, and *SUFU* (previously correlated with renal function in patients with Enterobacteriaceae bacteremia), but none reached genome-wide significance [50].

The limited number of GWAS in sepsis and the consistent failure to replicate most findings reflect a fundamental methodological constraint. Specially, sample sizes of studies are insufficient to achieve a robust statistical power required to detect the modest effect sizes expected for complex traits at genome-wide significance thresholds [36]. Additional challenges include clinical heterogeneity of cases, appropriate controls selection, and lack of population diversity representation beyond European genetic ancestry. Drawing controls from the general population may include individuals who have had or will develop sepsis, introducing a potential bias [46]. However, sensitivity analyses in other infectious diseases suggest that this may not substantially bias effect size estimates, as observed in COVID-19 [51]. The increasing sample size through large international consortia represents the most tractable path forward. The COVID-19 experience illustrates this as the use of population controls, facilitated the aggregation of over 200,000 cases and 3 million controls across biobanks, enabling the identification of 51 genome-wide significant loci and the mapping of three major biological pathways underlying susceptibility and severity [52].

### Polygenic predictors

Polygenic risk scores (PRS) aggregate effects of numerous genetic variants associated with a trait, weighting each according to its estimated effect size from GWAS, to generate an individual-level estimate of genetic predisposition [[53], [54], [55]]. Beyond individual risk stratification, PRS have proven useful for defining endotypes within heterogeneous patient populations, evidencing shared genetic architectures across traits, guiding preventive strategies, optimizing trial participant selection, and identifying novel therapeutic targets [56,57].

For sepsis, PRS models have been scarcely used. Engoren et al. [39] constructed PRS for Sepsis-2 and Sepsis-3 definitions from GWAS data in a predominantly European-ancestry cohort, demonstrating that aggregating the modest genetic effects discriminate sepsis patients from non-sepsis controls (c-statistic = 0.752 for both definitions). However, it remains unknown whether the PRS adds predictive value beyond routinely available clinical variables. Using a cross-trait PRS approach, D'Urso et al. [38] showed that PRS for CRP levels were associated with septic shock, while PRS for hematocrit and granulocyte count were associated with 28-day mortality, suggesting that genetic predispositions underlying sepsis susceptibility and progression are partially distinct. This was further supported by Hernandez-Beeftink et al. [46] who found that a PRS for sepsis susceptibility was not associated with survival outcomes at any significance threshold, and none of the previously reported sepsis mortality variants were replicated. Beyond acute risk, PRS and multi-trait genetic analyses hold promise for elucidating the shared pathogenic mechanisms between sepsis and ARDS, with potential implications for drug repositioning and novel therapeutic targets [58].

However, the translational potential of PRS in critical infectious illness has been most compellingly illustrated in COVID-19, where the rapid assembly of large international consortia enabled well-powered scores [51]. For example, individuals in the top decile of a genome-wide PRS had more than twice the risk of severe disease compared to those at median risk [59], and pathway analyses of COVID-19 PRS revealed enrichment of variants in immune-related pathways, including oligoadenylate synthetase antiviral response and IL-10 signaling, providing a mechanistic framework that directly informed therapeutic target identification [60,61]. COVID-19 PRS have also revealed genetic connections between acute infectious illness and chronic lung disease. A PRS for idiopathic pulmonary fibrosis was significantly associated with COVID-19 hospitalization and severe illness with shared variants enriched in cadherin and integrin signaling pathways, suggesting that genetic predisposition to chronic fibrotic lung disease may influence acute infection severity and post-infectious lung sequelae [62].

### From association to causation

A key challenge in sepsis biomarker research is that a circulating molecule or a genetic variant may be strongly associated with an adverse outcome without actually causing it. It is essential to distinguish biomarkers that causally contribute to disease from those that are correlated but causally unrelated, since only the former are rational therapeutic targets. Mendelian randomization (MR) is an analytical approach that uses germline genetic variants as instrumental variables to assess whether an observed association between a modifiable exposure, such as circulating protein levels, and a clinical outcome is causal [[63], [64], [65]]. The reliability of MR stems from genetic inheritance. Germline variants, fixed at conception and randomly allocated at meiosis, are free from the confounding and reverse causation that limit observational measurements. As a result, a genetic variant robustly associated with an exposure can act as a proxy. In sepsis, where many biomarkers of the acute inflammatory phase correlate with outcome, but lack clear causal roles, MR can prioritize those on the causal pathway, isolating the most promising therapeutic targets [66].

MR has been scarcely applied in sepsis. Two studies illustrate its application to sepsis-associated ARDS. Reilly and colleagues used MR and mediation analysis to study plasma ANG-2, a classical biomarker of vascular permeability [67]. They found that genetically predicted plasma ANG-2 levels (determined by *ANGPT2* variants) were significantly associated with ARDS risk in patients with sepsis, supporting its potential causal factor of ARDS development [67]. Jones and colleagues evaluated plasma sRAGE (soluble receptor for advanced glycation end products) as a causal intermediate of ARDS. Selecting genetic instruments as predictors of its levels, they estimated a consistent causal effect of plasma sRAGE on ARDS risk in two sepsis cohorts, supporting that elevated sRAGE causes ARDS [68]. Together, these two demonstrate that ANG-2 and sRAGE are causal intermediates and candidate therapeutic targets in sepsis-associated ARDS.

### Sequencing-based studies and the risk apportioned by rare variants

The genetic approaches discussed so far are based on common genetic variants, present in more than 1% of the population. However, common variants only explain part of the heritable sepsis susceptibility and its complications, and a complementary fraction may be contributed by rare variation [69]. Sequencing the genome or specific regions directly is required to capture this fraction of the allele frequency spectrum, motivating the use of next-generation sequencing (NGS) in critically ill populations, in whom both common and rare variants may influence immune responses, disease severity, and outcomes.

NGS allows sequencing millions of DNA fragments simultaneously, improving throughput, accuracy, and cost per base, while enabling the identification of genetic variants across the full spectrum of frequency and the affected sequence type [70,71]. Whole-genome sequencing (WGS) covers coding and noncoding sequence, while whole-exome sequencing (WES) targets the protein-coding fraction (〜1% of the genome), enriched in variants more likely to be pathogenic. Targeted panels restrict the application to phenotype-relevant genes [71]. Together, these approaches enable systematic assessment of genomic variation to prioritize candidate biomarkers.

In sepsis, WGS and WES offer a largely hypothesis-free strategy to uncover host genetic susceptibility not captured by conventional genotyping [70,71]. To date, no WGS study has been conducted in sepsis, and all sequencing cohort studies have relied on WES, mostly comprising limited numbers of cases and controls. One showed that the burden of rare deleterious variants in patients with sepsis, rather than any single gene, was predictive of the disease course. Rare variants in cell signaling and innate immunity pathways, notably Gαq signaling, Toll, and CDC42, discriminating favorable from adverse outcomes [72]. This suggests that the aggregate rare-variant burden may itself serve as a genomic marker of prognosis rather than pointing to a single causal gene. In a study of infectious purpura fulminans, an extreme phenotype of sepsis with coagulopathy, Bendapudi et al. [73] used pathway-based rare variant burden testing to identify a significantly increased load of low-frequency, putatively function-altering variants in the complement system. Functional characterization revealed that variants in the complement receptors CR3 (ITGAM/ITGB2) and CR4 (ITGAX/ITGB2) resulted in loss of anti-inflammatory CR3 function and/or gain of pro-inflammatory CR4 function, with each additional complement variant conferring risk for purpura fulminans after adjusting for age, sex and disease severity. In the largest WES sepsis study to date, rare variants fulfilling additional deleterious prediction criteria (qualifying variants), were aggregated into clusters assisted by the molecular interaction knowledge to address genetic heterogeneity [74]. The study identified nine biologically connected gene clusters enriched in distinct pathways associated with ARDS risk, most notably TLR cascades [74]. Taken together, these findings highlight that deleterious genetic variants can serve as genomic prognosis predictors, and the genes and pathways involved offer guidance for biomarker discovery.

Rare variant analysis can also influence the response to infection, as demonstrated in severe COVID-19 by the COVID Human Genetic Effort (CHGE). After linking rare variants in type I interferon immunity genes to life-threatening disease [75], Asano and colleagues focused on the X-linked gene *TLR7.* Combining WGS and WES, patient-cell assays, and family studies. They found very rare, deleterious *TLR7* variants in 16 of 1,202 critically ill men with COVID-19, but in none of 331 men with mild infection. Patients' cells failed to respond to *TLR7* stimulation and produced little type I interferon against SARS-CoV-2, and the variants were traced through sixteen families as predominantly inherited. This established X-linked *TLR7* deficiency as a highly penetrant cause of life-threatening SARS-CoV-2 infection in men under 60 (1.8% of cases), illustrating how sequencing can move from statistical association to demonstrated inherited causality [76]. NGS thus complements common variant-based approaches, establishing causality and identifying mechanistically based candidate biomarkers in sepsis.

**Fig. 1:**
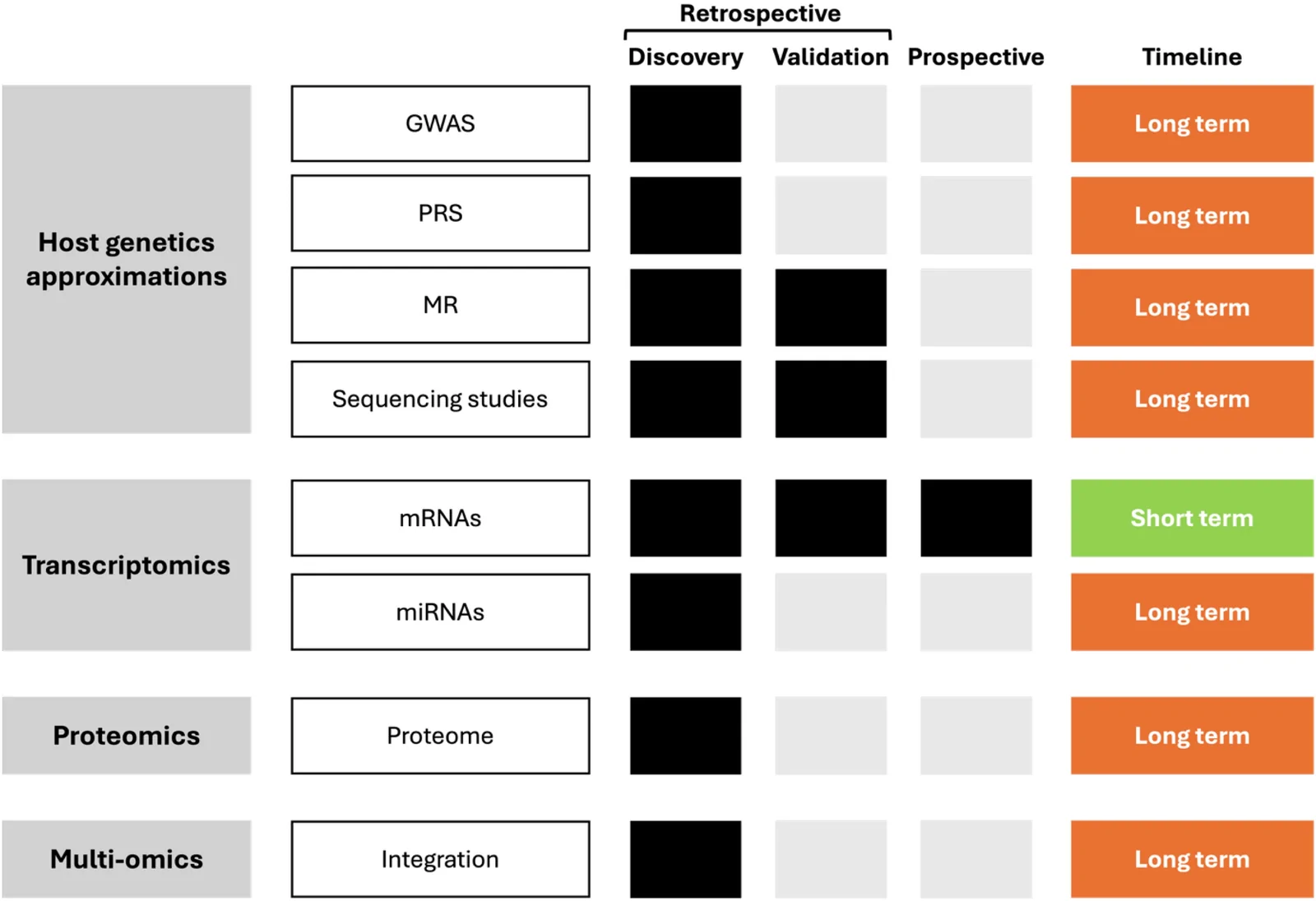
Molecular approximations and their translational potential for sepsis. Each approach was evaluated on three dimensions considering the literature of existing retrospective and prospective studies based on: i) evidence-supporting studies (Discovery); ii) studies in independent cohorts (Validation); and iii) prospective studies (Prospective). Black boxes represent the existence of published studies. A timeline column denotes whether the approach is considered short-term (green) or long-term for translation (orange) based on the published studies (For interpretation of the references to colour in this figure legend, the reader is referred to the web version of this article).

Regarding clinical translatability, knowledge from host genetics currently has yet limited direct application in precision sepsis management (Fig. 1). In the context of evidence of severe or recurrent infections, the most actionable genetic approach is a sequencing-based study, as it is nowadays used for inborn errors of immunity diagnosis [77]. A positive finding of a monogenic variant linked to the phenotype could imply a molecular diagnosis informing patient management. However, this is still far from the routine clinical practice in adult sepsis patients. While MR contributes guiding drug development by prioritizing causal therapeutic targets, it does not directly inform bedside decisions, as genetic evidence indicates what to target rather than whom to treat or when to intervene. In the long term, robust PRS models based on ancestry-diverse GWAS may enable genetic risk stratification among patients. However, current sample sizes of these studies, the modest discriminatory capacity of common genetic variants (in isolation or as part of PRS models), and the European-centric bias of studies imply that this is not yet feasible for clinical implementation.

## Transcriptomics

**Fig. 2:**
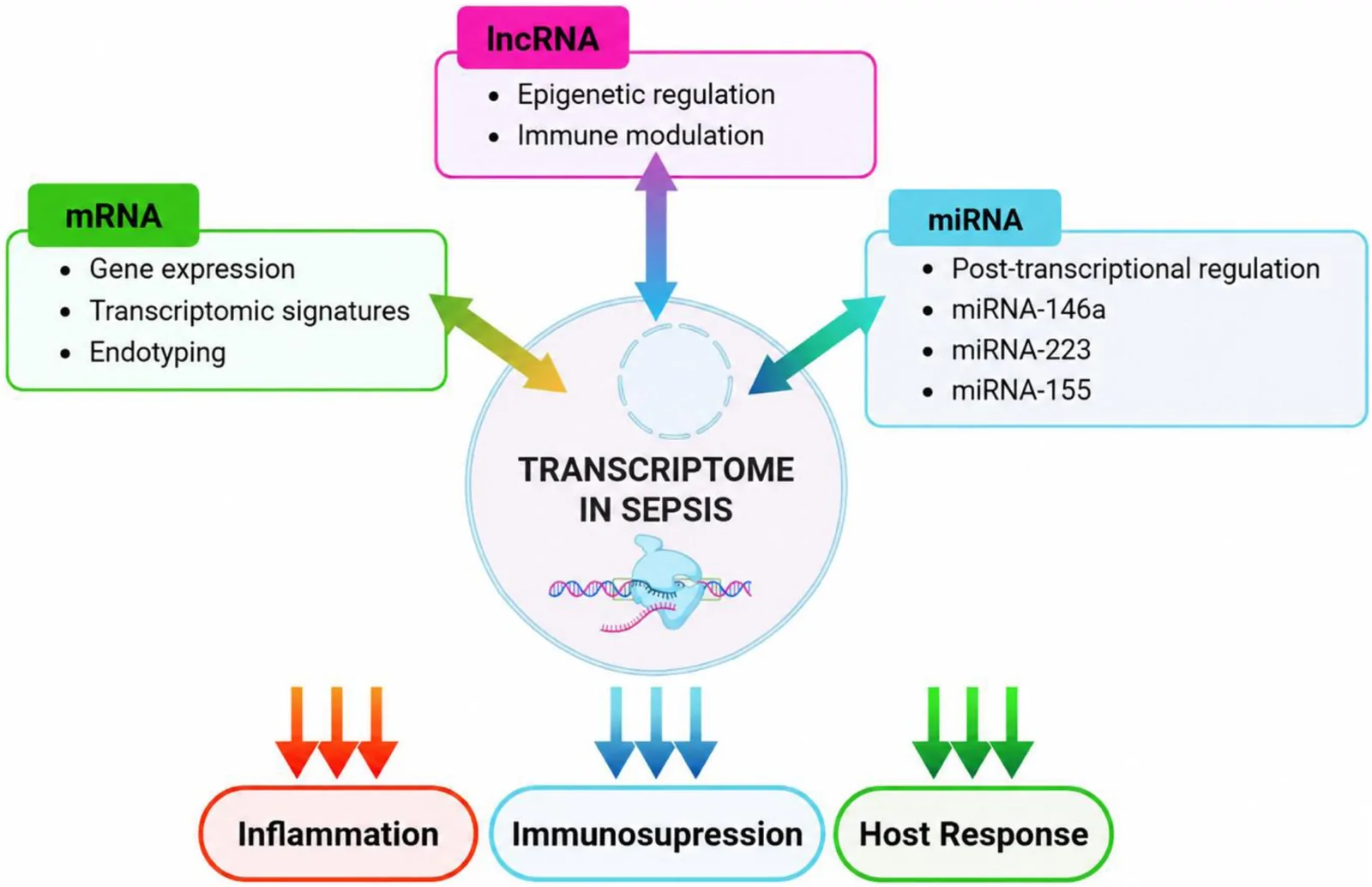
Schematic overview of transcriptomics components involved in sepsis. mRNA, messenger RNA; lncRNA, long non-coding RNA; miRNA, microRNA.

The transcriptome is the complete set of RNAs expressed in a cell or tissue under specific conditions and, unlike the relatively stable genome, it is highly dynamic and reflects cellular functional status [78]. It includes messenger RNAs (mRNAs) and regulatory non-coding RNAs (ncRNAs), such as miRNAs and long ncRNAs (lncRNAs) [79,80] and is primarily analyzed by RNA sequencing or microarray platforms to quantify global gene expression changes [78,80]. Much of this progress stems from whole-blood transcriptomics, which can predict sepsis subtypes (Fig. 2).

Prioritizing a single transcriptomic biomarker remains difficult, because these studies typically rely on coordinated over- or under-expression of gene sets rather than on isolated transcripts. Single-cell transcriptomic profiling with external validation identified S100A11 (calcium-binding damage-associated molecular patterns [DAMPs] that promotes inflammation via RAGE and TLR4), QPCT (an enzyme that stabilizes the monocyte chemoattractant CCL2 through pyroglutamate formation), and IFITM2 (an interferon-induced antiviral protein that enhances type I IFN signaling) as candidate biomarkers upregulated in immune cells during sepsis, with QPCT expressed mainly in macrophages and all three linked to prognosis [81].

Multi-gene signatures outperform individual transcripts for both diagnostic and prognostic purposes. For diagnostic discrimination, a five-gene signature (CMTM5, CETP, PLA2G7, MIA, and MPP3) differentiated sepsis from systemic inflammatory response syndrome (SIRS) in a multicenter ICU cohort [82], and the commercial Septicyte four-gene panel discriminates sepsis from infection-negative systemic inflammation [83]. A meta-analysis of more than 17,000 patients showed that Septicyte transcriptomic signatures outperformed CRP and procalcitonin in distinguishing sepsis from SIRS [84].

### mRNA transcriptomics

For prognostic stratification, several mRNAs transcriptomic endotyping systems have been developed. Davenport et al. [85] defined two sepsis response signatures (SRS): SRS1, characterized by immunosuppression, HLA class II downregulation, and higher mortality; and SRS2, reflecting a distinct immune state. These signatures exposed sepsis heterogeneity and revealed patient subgroups with different prognoses. This concept was later refined into the quantitative immune dysfunction score SRSq [86], derived from whole-blood transcriptomes and measurable from a reduced 7–12 gene signature, which stratifies infection severity and predicts outcomes. Scicluna et al. [87] identified four Molecular Diagnosis and Risk Stratification of Sepsis endotypes (designated as MARS1 to MARS4). Patients in MARS1 associated with immunosuppression and had the worst survival; MARS2 to MARS4 engaged different pathways of the immune response and were associated with better survival. On the other hand, Sweeney et al. [88] described three robust endotypes across 23 international datasets: inflammopathic (innate activation), adaptative (adaptative immunity), and coagulopathic. In this context, Sinha et al. [89] validated hyper/hypoinflammatory phenotypes, originally defined in ARDS [90], showing differential response to activated protein C. This integrated host-microbe approach was pioneered by Langelier et al. in critically ill adults with lower respiratory tract infections [91] and extended to sepsis diagnosis using plasma metagenomics [92]. Neyton et al. [93] took this evidence to a new level by integrating host-microbe blood metagenomics, and confirming the overlap between these phenotypes and both SRS and MARS frameworks, with increased Enterobacteriaceae associated with the hyperinflammatory phenotype. More recently, a consensus framework of three blood consensus transcriptomic subtypes (CTS) was defined [94], each characterized by distinct activation of inflammatory, heme metabolism, or interferon signaling pathways. This framework was robust across diverse cohorts and informative for therapeutic decisions such as corticosteroid use.

### miRNA transcriptomics

Among ncRNAs, miRNAs regulate post-transcriptional gene expression by modulating the translation of specific mRNAs [95]. The most prominent circulating miRNAs in sepsis are miR-146a and miR-223, typically downregulated, and miR-155, which enhances the inflammatory response [[95], [96], [97]]. Extracellular vesicle-derived miRNAs from whole blood have also emerged as promising biomarkers for differentiating septic from non-septic shock, while hsa-miR-150-5p shows differential expression between septic shock and sepsis patients [98,99].

Transcriptomics is the closest to clinical translation in sepsis (Fig. 1). In the short term, mRNA transcriptomic endotyping could guide immunomodulatory therapy selection, as demonstrated by the differential response to corticosteroids by SRS endotypes [100] or the hyper/hypoinflammatory phenotype groups [89]. Commercially available targeted blood-based mRNA transcriptomic solutions already support sepsis diagnosis and the likelihood of a bacterial or viral infection [101], making endotype assignment technically feasible. miRNA profiling and dynamic transcriptomic monitoring with serial profiling could enable real-time treatment adjustments in the future. However, this would require faster turnaround times, lower costs, and prospective validation in endotype-stratified trials for it to be adopted broadly by the community.

## Proteomics

Protein biomarkers have been used for early detection of infection and sepsis. However, their individual ability to predict disease progression and outcomes are limited. The proteome is the complete set of proteins expressed by a cell, tissue, or organism at a given time, and proteomics is the large-scale study of their expression, structure, function, and interactions [102]. Because proteins are the principal effectors of cellular function with abundance governed by translation, post-translational modification, secretion, and degradation, proteomics offers a direct, functional view of the active state of cells and tissues [103,104].

In sepsis, the plasma proteome is the most accessible window onto the systemic response [105], given the dysregulation across coagulation, complement, and immune-effector pathways. However, it is also one of the most demanding analytical challenges, spanning over ten orders of magnitude in protein concentration [106].

At the broadest level, proteomics enables the biological heterogeneity of sepsis to be categorized into clinically meaningful endotypes. Two complementary analytical strategies are used: discovery mass spectrometry (MS) (i), providing untargeted, broad-scale profiling by identifying and quantifying thousands of proteins simultaneously, and targeted (ii), panel-based assays, which measure predefined protein sets with higher throughput and reproducibility. A recent study applied MS to analyze 609 plasma proteins in a German multicenter cohort of adults with sepsis [107] using unsupervised clustering to define four molecular endotypes that differed markedly in severity. These ranged from a baseline profile to a most-severe cluster with a 100% mortality rate. The endotypes were characterized by distinct immunological features, including differential immunoglobulin abundance and acute-phase inflammation. A targeted approach yielded a convergent picture: profiling 92 inflammatory proteins in adults with severe infection, two inflammatory endotypes were identified by hierarchical clustering, with the highly inflammatory endotype present in older patients with lower lymphocyte counts [108]. The independent resolution of high- and low-inflammatory patient strata using both untargeted and targeted proteomics supports that protein-defined endotypes capture a robust axis of host response differentiation relevant to patient stratification.

For sepsis prognosis, MS-derived multi-protein panels have outperformed individual analytes, and adding a temporal dimension increases their value. In a single-center cohort of adults with sepsis and septic shock, discovery plasma proteomics identified a nine-protein signature predicting organ dysfunction (GPX3, APOB, ORM1, SERPINF1, LYZ, C8A, CD14, APOC3, and C1QC) and a separate 22-protein signature predicting mortality [109]. Longitudinal designs provide further resolution. In a multicenter sepsis cohort, plasma proteome dynamics determined by MS profiling between days 1 and 4, were dominated by coagulation, immune, and complement pathways, and were associated with 30-day mortality [110]. These studies suggest that the patient’s proteome trajectory, rather than a single snapshot, contributes prognostic information.

Regarding sepsis complications, targeted proteomic profiling has helped resolving sepsis-associated AKI, one of its most frequent and lethal manifestations [111]. Measurement of plasma H3.1 nucleosomes, a neutrophil extracellular trap component quantified by a targeted immunoassay, in a large critically ill adult cohort, tracked organ failure and a hyperinflammatory endotype predicting the need for renal replacement therapy [112]. This signature complements the broader organ-dysfunction panel [109], demonstrating how the proteome can address both global multi-organ dysfunction and, the AKI that frequently complicates sepsis.

**Fig. 3:**
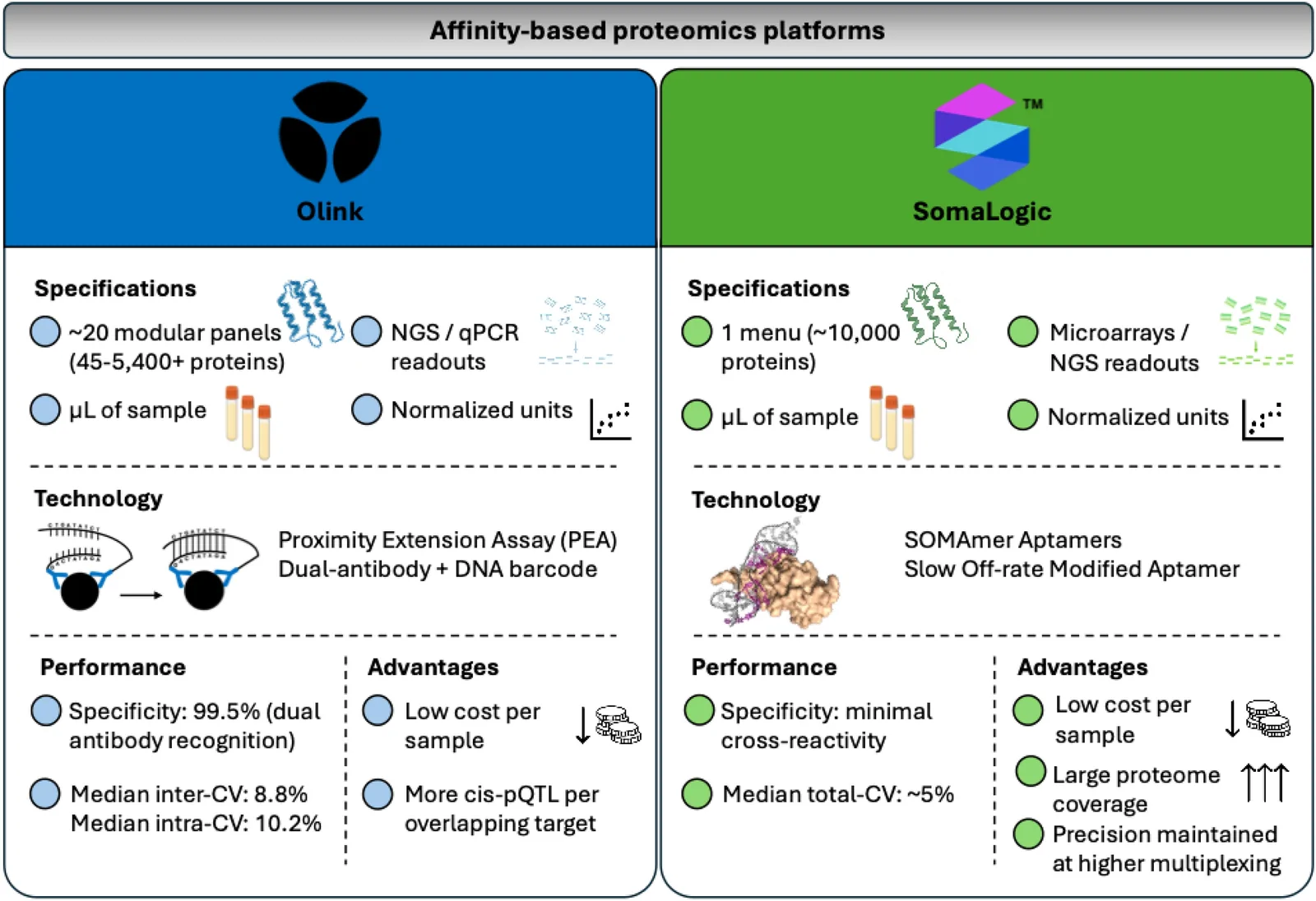
Comparison of the two main affinity-based proteomics platforms (Olink and SomaLogic) used in large-scale biomarker discovery. NGS, next generation sequencing; qPCR, quantitative polymerase chain reaction; CV, coefficient of variation; cis-pQTL, cis-acting protein quantitative trait loci.

Although these approaches help to identify potential biomarkers and sepsis endotypes, other NGS-based protein detection methods are becoming popular. Affinity-based platforms such as Olink and SomaLogic (Fig. 3), provide relative quantification of targeted protein panels with high reproducibility and throughput, offering a complementary route to confirm candidate biomarkers and discover stratifying signatures within the panel's content [113]. Studies relying on these approaches remain limited in sepsis. The clearest demonstration of their potential for infectious diseases comes from COVID-19, as a model of viral sepsis [114]. Parke and colleagues [115] used the affinity-based Olink Explore platform targeting nearly 1500 proteins in hospitalized adults with COVID-19, linking disease severity to a neutrophil-centered inflammatory proteomic signature (notably calprotectin and S100A12). This provided a proof-of-concept for the type of targeted panel-based stratification that could be deployed in bacterial sepsis.

From a translational perspective, proteomics provides a simple and accessible route to clinical implementation (Fig. 1). Individual protein biomarkers (CRP, PCT) are already embedded in clinical practice leveraging classical ELISA measurements. Plasma biomarker-based phenotyping could be adapted for stratification using routinely measurable analytes directly from peripheral blood in the long term. MS-based and affinity-platform proteomics may help to define targeted proteins defining endotypes for therapy selection. However, current evidence in sepsis remains still insufficient for bedside application.

## Multi-omics

Multi-omics approaches systematically integrate multiple layers of biological information, such as genomics, transcriptomics, and proteomics, among others, to obtain a comprehensive view of biological systems [116,117]. Unlike single-layer omics approaches, multi-omics captures interactions across different levels of biological regulation, enabling a deeper interpretation of the pathophysiological mechanisms underlying disease [116,118]. This integrative strategy has become a cornerstone of precision medicine, where heterogeneous data combined with machine-learning tools allows the identification of robust clinically relevant biological patterns [[116], [117], [118]].

In sepsis, the complexity of the host-pathogen interaction and marked interindividual variability in immune and metabolic response limit the utility of single-layer biomarkers. Integrating multiple omics layers can improve understanding of pathogenic mechanisms and identification of more precise molecular signatures for diagnosis, patient stratification, personalized therapy, and prognosis [119]. A representative example is the integration of host transcriptomics and plasma proteomics in adult patients with sepsis from the Functional Genomics in Severe Infections (FUSE) project, which defined an interferon-dependent inflammatory endotype characterized by a coordinated IFN-γ–CXCL9–CXCL10 axis [120]. Combining transcriptional and protein-level information resolved a host-response endotype that neither layer captured fully on its own, illustrating how data integration refines patient stratification beyond single-omics signatures. Similarly, integrating genotyping and RNA-sequencing data from 638 adult sepsis patients in the UK Genomic Advances in Sepsis (GAinS) study, 16,049 expression quantitative trait loci were mapped and identified significant interactions between genotype and the SRS subgroups for 1,578 SNP-gene pairs, linking host genetic variation to regulatory networks and cell subtypes underlying transcriptomic endotypes [121]. However, multi-omics studies in sepsis remain scarce, and most available evidence derives from single-omics analyses or COVID-19 cohorts framed as models of viral sepsis.

Beyond stratification, multi-omics approaches have been applied to biomarker discovery. In a study integrating transcriptomic endotyping with eight protein-based biomarkers in 167 sepsis patients with associated AKI, combining endotype classification with suPAR or bio-ADM achieved optimal predictions of kidney replacement therapy or death, outperforming either layer alone [122]. A comparative multi-omic analysis of COVID-19 ARDS and bacterial sepsis-induced ARDS quantified 1,980 molecules across metabolomic, lipidomic, and proteomic platforms, identifying 706 differentially abundant molecules and subnetwork-based signatures associated with clinical outcomes [123]. In a four-omics integration of transcriptomic, proteomic, metabolomic, and lipidomic data from 128 COVID-19 patients, 219 molecular features were associated with disease severity, including dysregulation of complement activation, lipid transport, and neutrophil degranulation [124].

Multi-omics approaches represent a powerful strategy for biomarker discovery in sepsis, capturing the complexity across multiple biological dimensions. However, these approaches face significant and shared challenges, including data heterogeneity, high dimensionality, and the need for advanced computational methods for integration and interpretation, that currently constrain their translation into clinical practice [116,119].

**Fig. 4:**
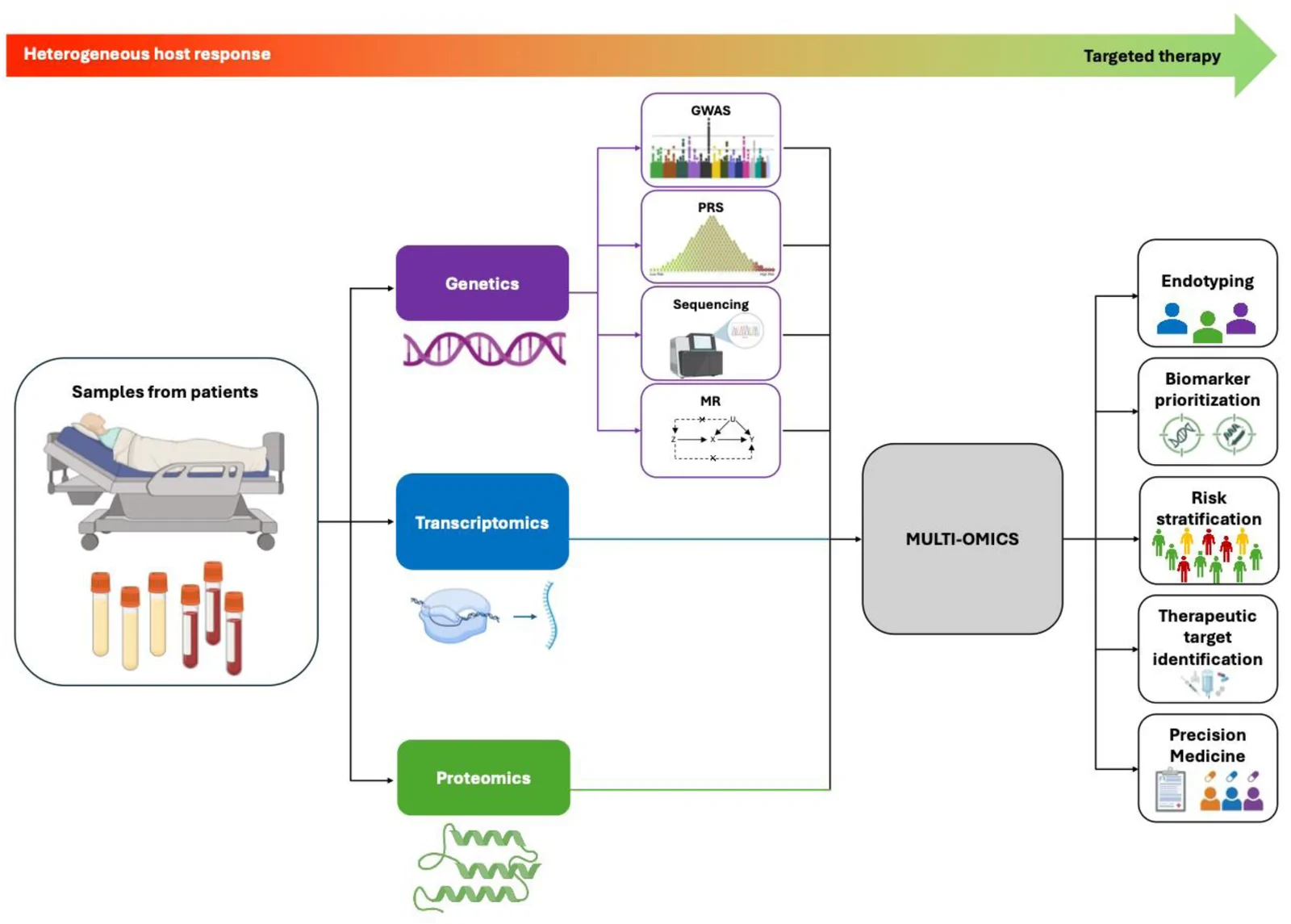
A framework for multi-omics integration and precision medicine in sepsis and critical illness. Starting from patients with sepsis, characterized by a heterogeneous host response, or from critical illness, the analysis of multiple omics layers (genetics, transcriptomics, and proteomics) enables endotyping, biomarker prioritization, risk stratification, and therapeutic target identification, collectively converging on precision medicine and targeted therapy. GWAS, genome-wide association studies; PRS, polygenic risk scores; MR, Mendelian randomization. The PRS figure is courtesy of the National Human Genome Research Institute (https://www.genome.gov).

Among multi-omics integration strategies, combining genome-wide association data with circulating proteomics is emerging as a particularly relevant approach in sepsis, as it enables the identification of disease-associated loci and causal inference through MR and therapeutic target prioritization, extending the scope of current multi-omics applications, which in sepsis have largely focused on associative analyses. GWAS advances, together with the high-throughput proteomic approaches sustain proteogenomics [113,[125], [126], [127]], which lies at the intersection of genomics, transcriptomics, and proteomics, improving the functional interpretation of genetic information (Fig. 4). Proteogenomics inform of protein quantitative trait loci, genetic variants associated with protein levels [125] providing biologically informative links between genetic variation and downstream molecular phenotypes, revealing candidate therapeutic targets [113,128,129].

In sepsis, proteogenomics is emerging as a promising strategy. By combining data from five large cohorts, plasma apolipoprotein E (ApoE) was identified as a causal factor for sepsis susceptibility [130]. Experimental validation in murine models demonstrated that both ApoE deficiency and excess exacerbate inflammation, organ injury, and mortality. Similarly, a proteogenomic analysis of circulating SERPINA1 levels (which encode alpha-1 antitrypsin) supported a causal association between higher genetically predicted SERPINA1 levels and lower risk of sepsis, suggesting that an altered protease-antiprotease balance contribute to pathogenesis [47]. Two proteogenomic studies have prioritized *PTPRD* [131] and *TCF20* [132] as novel candidates for sepsis-associated ARDS risk. Ultimately, these studies demonstrate the clinical utility of proteogenomic profiling for risk stratification, highlighting the need to distinguish between mere correlations and true mechanistic pathways in sepsis outcomes.

From a translational standpoint, multi-omics integration remains a long-term endeavor (Fig. 1) since no multi-omics signature has yet been validated for use at the bedside in cases of sepsis. For now, the technical complexity and long waiting times are incompatible with the rapid decision-making required in acute care.

## Future perspectives

Despite substantial progress, clinical translation of omics biomarkers in sepsis remains limited. Sepsis is a clinically heterogeneous syndrome, and the absence of standardized protocols for sample collection, processing, and analysis introduces marked technical variability across cohorts [133,134]. Many studies have relied on small adult European single-center cohorts limiting generalizability and robustness. Thus, although the literature is plagued by candidate biomarkers, most lack formal validation [13]. Large biobank-scale analyses partially address sample size. However, introduce other limitations because case and control definitions rely on electronic health records lacking deep clinical characterization. Population controls may have experienced sepsis, or may develop it in the future, blurring the contrast under study [135].

As discussed throughout this review, the readiness of each approach for clinical translation differs (Fig. 1). In the short term, the most directly applicable approaches include transcriptomic endotyping to stratify immunomodulatory therapy and host-response gene expression panels to diagnose sepsis and infection. In the long term, advances that require further evidence include dynamic, longitudinal, multi-omics profiling, prospective randomized trials that are stratified by endotype, the integration of host and microbial sequencing, and artificial intelligence-assisted multi-omics decision support. This distinction reflects the maturity of clinical evidence rather than technological capability. Approaches with demonstrated differential treatment responses are ready for confirmatory testing, whereas multi-omics integration and computational prediction require fundamental validation.

Some biomarkers are also dynamic and context-dependent. Proteomic signatures vary according to sampling time and patient state. Reliance on blood sampling means that other potentially informative cell-type- and tissue-specific signals could be overlooked [134]. Beyond these constraints, several emerging layers are expanding our understanding of sepsis at a molecular level. The transition from early hyperinflammation to immunosuppression is underpinned by epigenomic regulation, DNA methylation, histone modifications, and chromatin organization [136], and can profiled using methylation arrays and sequencing-based methods. Although epigenetic studies in adults with sepsis are emerging, including epigenome-wide analyses linking leukocyte methylation to immunosuppressive phenotypes [137], they remain few and small, and no epigenetic biomarker has been validated for clinical use. Metabolomics provides a functional, time-resolved readout of the host functional state that remains underutilized for patient subtyping [138].

Metagenomics reframes the concept of biomarkers from single pathogens towards integrated microbial signatures, with cell-free DNA and metagenomic NGS improving pathogen detection where cultures fail [139]. This host-microbe integration pioneered by Langelier et al., combining pathogen detection, airway microbiome profiling, and host transcriptomic signatures in critically ill adults [91], was later extended to sepsis diagnosis via plasma metagenomics [92]. A concrete prognostic value is emerging since lung bacterial dysbiosis has been associated with ICU mortality in extra-pulmonary sepsis [48], and tracheal-aspirate metagenomics has linked antibiotic-resistance signatures to mortality in non-pulmonary sepsis [140]. Across all these molecular layers, artificial-intelligence and machine-learning methods are increasingly used to integrate multi-omics data and define endotypes through clustering. However, limited mechanistic interpretability and insufficient external validation still curb their clinical impact [141,142].

In summary, recruitment of larger, better characterized and more homogeneous longitudinal cohorts are required to capture the evolving host response. This must be done as part of international consortia that harmonize protocols and prospectively validate biomarkers, as well as rapid point-of-care platforms. A key priority will be deriving proteomic measurements from critically ill patients themselves, since instruments from general population cohorts may miss protein regulation specific to sepsis and provide misleading causal estimates [132]. The ultimate clinical goal remains to develop a simple, compact panel of validated biomarkers suitable for routine clinical use. Despite some promising signs from transcriptomic endotyping, this goal remains out of reach for now.

## Conclusions

The molecular characterization of sepsis is steadily shifting the field away from single, non-specific markers towards a biology-driven, integrated understanding of the host response. Multi-omics profiling is improving our ability to predict outcomes and, more importantly, to address the clinical heterogeneity that has long hindered therapeutic progress, by identifying endotypes characterized by different pathophysiology and prognosis. The central challenge now is to translate these signatures into robust, prospectively validated tools that perform reliably in adult patients across diverse settings. Early biomarker-guided trials have begun to demonstrate that endotype-stratified therapy is feasible, although scaling this approach still requires the multi-omic biomarker validation described in this review. Achieving this would transform sepsis care, moving it beyond a one-size-fits-all approach towards precision medicine, where patients would be stratified by their underlying molecular profile and matched to the appropriate intervention at the right time, ultimately improving survival rates for this heterogeneous and life-threatening syndrome.

## Authors’ contributions

Conceptualization: CF; Investigation: SGB, ESP, MBC, ET; Data curation: SGB, THB, CF; Writing - original draft: SGB, THB, CF; Writing - review & editing: all authors; Visualization: SGB, MBC; Funding acquisition: THB, SGB, CF.

## Declaration of Generative AI and AI-assisted technologies in the writing process

During the preparation of this work the authors used ChatGPT (GPT-4, version 1.2026.160, OpenAI) and Biomni (Phylo) in order to improve the English language and readability of the manuscript and assist with summarizing and synthesizing the reviewed literature. After using this tool/service, the authors reviewed and edited the content as needed and takes full responsibility for the content of the published article.

## Funding

ERA PerMed (JTC_2021; AC21_2/00039 from Instituto de Salud Carlos III) and funds from Next Generation EUas part of the actions of the Recovery Mechanism and Resilience; Instituto de Salud Carlos III (PI23/00980, CB06/06/1088, CD25/00028, FI24/00316) co-funded by the European Regional Development Funds, “A way of making Europe” from the EU; Fundación Canaria Instituto de Investigación Sanitaria de Canarias (PIFIISC23/05); Cabildo Insular de Tenerife (CGIEU0000219140 andCGIAC0000014697); Instituto Tecnológico y de Energías Renovables agreement (OA23/043).

## Declaration of competing interest

None.
